# Genetic diagnosis of three intellectually disabled individuals in a pedigree and insights into fragile X syndrome diagnosis

**DOI:** 10.3389/fnins.2025.1656418

**Published:** 2026-01-21

**Authors:** Jianmei Huang, Bing Kang, Xiaoliang Xia, Zhenglong Guo, Yibing Lv, Wenke Yang, Chenyang Wang, Jinming Wang, Shixiu Liao

**Affiliations:** 1 Institute of Medical Genetics, Henan Provincial People's Hospital, Zhengzhou University People’s Hospital, Zhengzhou, China; 2 National Health Commission Key Laboratory of Birth Defects Prevention, Henan Provincial Key Laboratory of Genetic Diseases and Functional Genomics, Zhengzhou, China

**Keywords:** fragile X syndrome, genetic counseling, genetic diagnosis, genetic testing, intellectual disability

## Abstract

Fragile X syndrome (FXS) is the most common inherited cause of intellectual disability (ID). However, its diagnostic rate needs to be improved by screening for specific populations. Here, we determined the genetic cause of three ID patients in the affected pedigree and derived diagnostic insights for FXS. Enrolled at Henan Provincial People’s Hospital in April 2025, the family underwent multiple diagnostic tests. Whole-exome sequencing failed to detect causative variants—consistent with its inability to identify dynamic trinucleotide repeat expansions. Expanded pedigree analysis showed the inheritance did not fit typical autosomal dominant/recessive or X-linked models. This raised suspicion of FXS. Trinucleotide repeat primed PCR with capillary electrophoresis (TP-PCR/CE) confirmed proband III-1 as an FXS full-mutation individual, and comprehensive FXS analysis (CAFXS) validated this result while identifying counselee III-2 as a female pre-mutation carrier. All three ID cases harbored *FMR1* full-mutation, with ID severity correlating with CGG repeat length. Notably, the maternal pre-mutation carrier (152 CGG repeats) had offspring with variable repeat dynamics: full-mutation (427 repeats) and reduced pre-mutation (71 repeats in III-2). These findings confirm FXS as the ID etiology and emphasize the clinical necessity of FXS-targeted screening in ID families with atypical inheritance patterns.

## 1 Introduction

Intellectual disability (ID), a neurodevelopmental disorder imposing significant psychological and economic burdens on families and society, has a global prevalence of 1–3% (Bustos and Findlay, 2023; Nair et al., 2022). X-linked intellectual disability (XLID) accounts for approximately 5–15% of all ID cases (Bustos and Findlay, 2023). Fragile X syndrome (FXS, OMIM 300624), the leading cause of XLID, represents ~50% of XLID cases and is the most common monogenic cause of inherited ID (Fernández et al., 2015). FXS follows an X-linked semidominant inheritance pattern, driven by abnormal expansion and methylation of CGG trinucleotide repeats in the 5′ untranslated region (5’UTR) of the fragile X messenger ribonucleoprotein 1 (*FMR1*) gene (Hagerman and Hagerman, 2021). This abnormality disturbs the expression of fragile X messenger ribonucleoprotein (FMRP), encoded by *FMR1*, resulting in various disorders of FXS, including neural development (Rodriguez et al., 2020; Sirois et al., 2024).

FXS is classified into four types based on the *FMR1* CGG repeat length: normal (≤44 repeats), intermediate (45–54 repeats), pre-mutation (55–200 repeats), and full-mutation (>200 repeats) (Spector et al., 2021). Individuals with full-mutation exhibit marked phenotypic heterogeneity: more than 99% of males and ~50% of females manifest with FXS, with females having milder symptoms (Clinical Genetics Group of Medical Geneticist Branch of Chinese Medical Doctor Association et al., 2022). Among pre-mutation carriers, 40–75% of males and 16–20% of females develop fragile X-associated tremor/ataxia syndrome (FXTAS) (Hagerman and Hagerman, 2016), while 20% of females are also at risk for fragile X-associated primary ovarian insufficiency (FXPOI) (Jacquemont et al., 2007; Shelly et al., 2021). Female pre-mutation carriers have the CGG repeat expansion risk, which results in transmitting full-mutation to offspring; this risk rate is as high as 100% when the CGG repetition number is more than 140 (Nolin et al., 2003). Thus, preconception and prenatal FXS screening exhibit valuable clinical significance in offering reproductive decisions to individuals with abnormal CGG repeats of *FMR1* and reducing FXS incidence.

Here, we identified that the three ID individuals in this study were suffering from *FMR1* full-mutation through multiple approaches—whole-exome sequencing (WES), triplet-repeat primed PCR (TP-PCR) with capillary electrophoresis (CE), and long-range PCR (LR-PCR) with long-read sequencing (LRS) (Liang et al., 2022). In addition, four pre-mutation carriers, including the counselee (III-2), were also detected. Furthermore, we discussed insights into the genetic diagnosis and counseling of FXS based on this pedigree.

## 2 Materials and methods

### 2.1 Participants

This study presents the results of a pedigree with three individuals with ID. The counselee (III-2), a 20-year-old female, sought preconception consultation at the Henan Provincial People’s Hospital in April 2025. Peripheral blood samples of 11 members in this pedigree were collected with informed consent for further study.

### 2.2 DNA extraction and sequencing

Peripheral venous blood (2 mL) was collected from study participants and anticoagulated with EDTA. Genomic DNA was extracted using a nucleic acid extraction kit (G3633, Servicebio, Wuhan, China). DNA concentration was quantified by the Nanodrop OneC spectrophotometer (Thermo Scientific, USA), and samples were stored at −20 °C for subsequent analysis.

For WES, Illumina libraries were constructed using the AIExomeV2 kit (iGeneTech Co., Beijing, China). Post-quality control (Roy et al., 2018), sequencing data were mapped to the human reference genome (hg19) via Burrows-Wheeler Aligner (v0.7.17) (Li and Durbin, 2009). Duplicate reads were removed using Picard (v1.128). Verita Trekker® Variants Detection System by Berry Genomics and the third-party software GATK (do Valle et al., 2016) were employed for variant calling. Variant annotation and interpretation were conducted by ANNOVAR (Wang et al., 2010) and the Enliven® Variants Annotation Interpretation System authorized by Berry Genomics. Single-nucleotide variants (SNVs) and small insertions/deletions (InDels; <30 bp) in protein-coding regions were analyzed, with a coverage of at least 98.97% at a 20 × depth threshold.

Fluorescent TP-TCR was performed using an *FMR1* gene CGG repeat detection kit (batch number: 210506, Microread Genetics Co., Beijing, China). Following capillary electrophoresis separation by 3500XL Dx genetic analyzer (Applied Biosystems, USA), *FMR1* CGG repeat counts were analyzed via GeneMapper software (Applied Biosystems, USA) according to manufacturer protocols.

Family samples underwent comprehensive analysis of fragile X syndrome (CAFXS) testing at Berry Genomics (Beijing, China). The exon1, exons 2–9 and 10–17, and the 997.4-kb upstream and 999.0-kb downstream flanking regions of *FMR1* gene were amplified using multiple probes. These amplicons were ligated with unique PacBio barcoded adapters, followed by SMRTbell library construction and sequencing for 30 h in circular consensus sequencing (CCS) mode. High-quality CCS reads were debarcoded and aligned to hg38 using the SMRT Link analysis software suite (Pacific Biosciences). Kernel density estimation method was applied to identify peaks from CCS reads, and the peaks were correlated to different alleles. The number of CGG repeats in each allele was determined by the read length. Due to amplification bias among different alleles and generally lower read yield for male samples with full-mutation, a result-based quality control of read number was used. For samples with only full-mutation alleles identified by kernel density estimation, CCS read number should be ≥100 for making a call; for samples with other results, CCS read number should be ≥2000 for making a call. For analysis of titrated mosaic samples, 2000 reads were randomly selected to determine the analytical sensitivity. The intragenic SNVs and indels of *FMR1* were identified by FreeBayes v.1.3.4 using ≥30 CCS reads. Large gene deletions were called in samples with over 30 on-target CCS reads from a plus reaction that had both forward and backward probes of the gap sequences on each end.

### 2.3 Pathogenicity interpretation of variants

Variants were interpreted for pathogenicity according to the American College of Medical Genetics and Genomics (ACMG) guidelines and those specific to FXS testing standards (Monaghan et al., 2013; Richards et al., 2015; Spector et al., 2021).

### 2.4 Statistical analysis

Per the manufacturer’s instructions, for FXS detection via TP-PCR/CE, the number of CGG repeats was calculated using the formula: *x* = (y − 229.21)/2.8406, where x represents the number of CGG repeats and y denotes the effective fragment size of the full-length peak. The frequency distributions of CGG repeat counts and AGG insertion patterns were presented.

## 3 Results

### 3.1 Clinical features of the family

**Figure 1:**
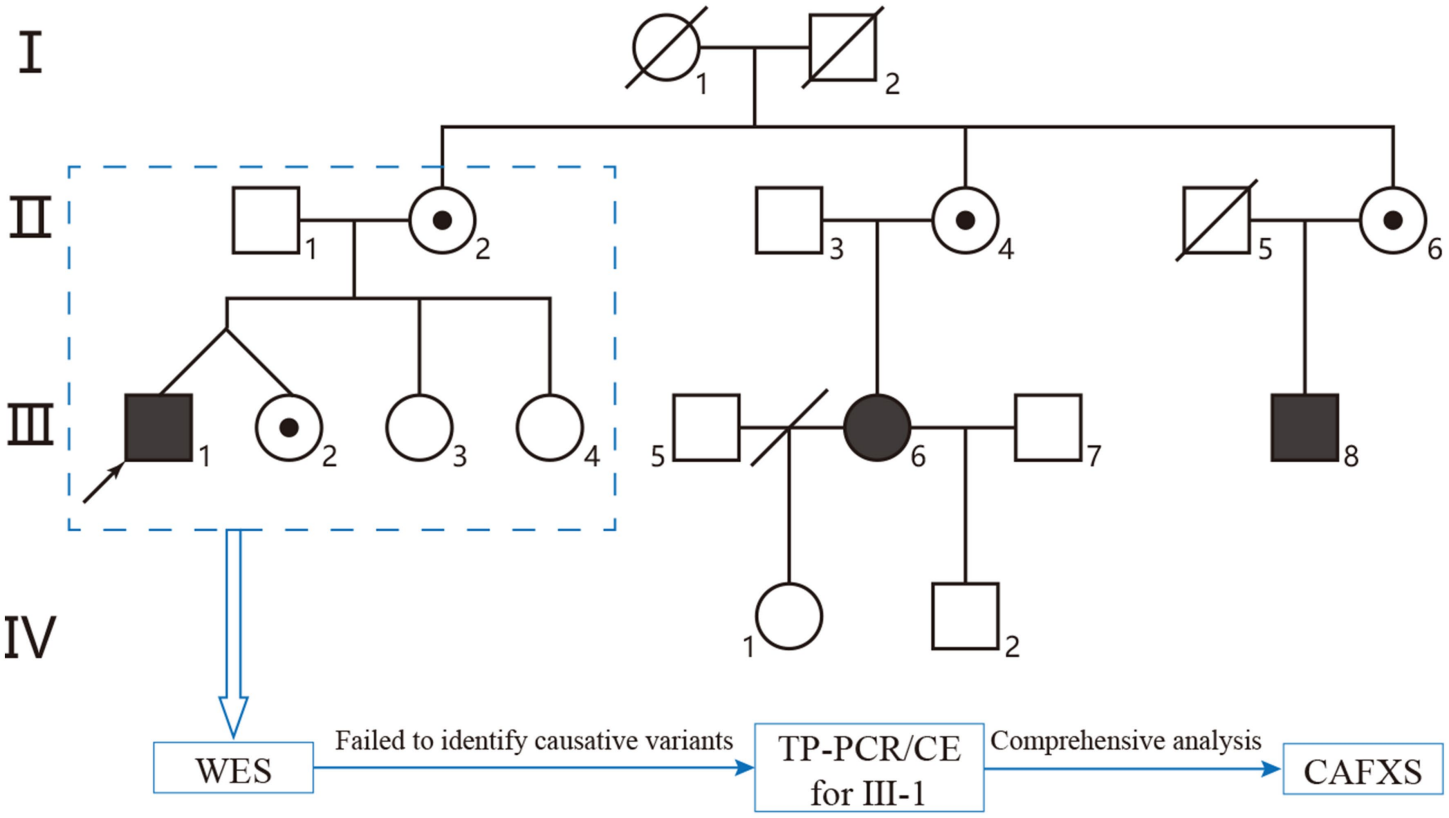
The pedigree chart and diagnostic strategies of this study. Males and females in the pedigree are represented by squares and circles, respectively. Shaded symbols indicate affected individuals, and dots inside symbols denote carriers. The black arrow represents the proband.

The pedigree enrolled in this study included three individuals with ID of varying severities: the counselee’s brother (proband III-1), cousin III-6, and cousin III-8 (Figure 1). Proband III-1 exhibited the most severe ID, accompanied by irritability, aggressive behavior, and protruding ears. III-6 had the mildest ID and gave birth to two asymptomatic children with different partners (Figure 1). III-8 had ID severity intermediate between III-1 and III-6, along with a long face and protruding ears. The counselee (III-2) presented with irregular menstruation, and baseline hormone levels showed normal follicle-stimulating hormone (FSH).

### 3.2 WES failed to identify causative variants

At the first visit, counselee III-2 only provided clinical information of six individuals that were her parents (II-1 and II-2), her brother (proband III-1), and her sisters (III-3 and III-4) (shown in blue dotted-line box in Figure 1). Peripheral blood karyotype analysis of counselee III-2 showed no positive results (data not shown). Trio-WES revealed no disease-causing variants matching the family phenotype.

### 3.3 TP-PCR/CE revealed the proband was FXS

When informing counselee III-2 of the negative WES results, we emphasized the importance of family history and clinical phenotypes in genetic counseling and diagnosis. This prompted counselee III-2 to supplement medical history data, revealing two additional ID cases in her maternal aunt’s family—totaling three ID individuals in this pedigree.

**Figure 2:**
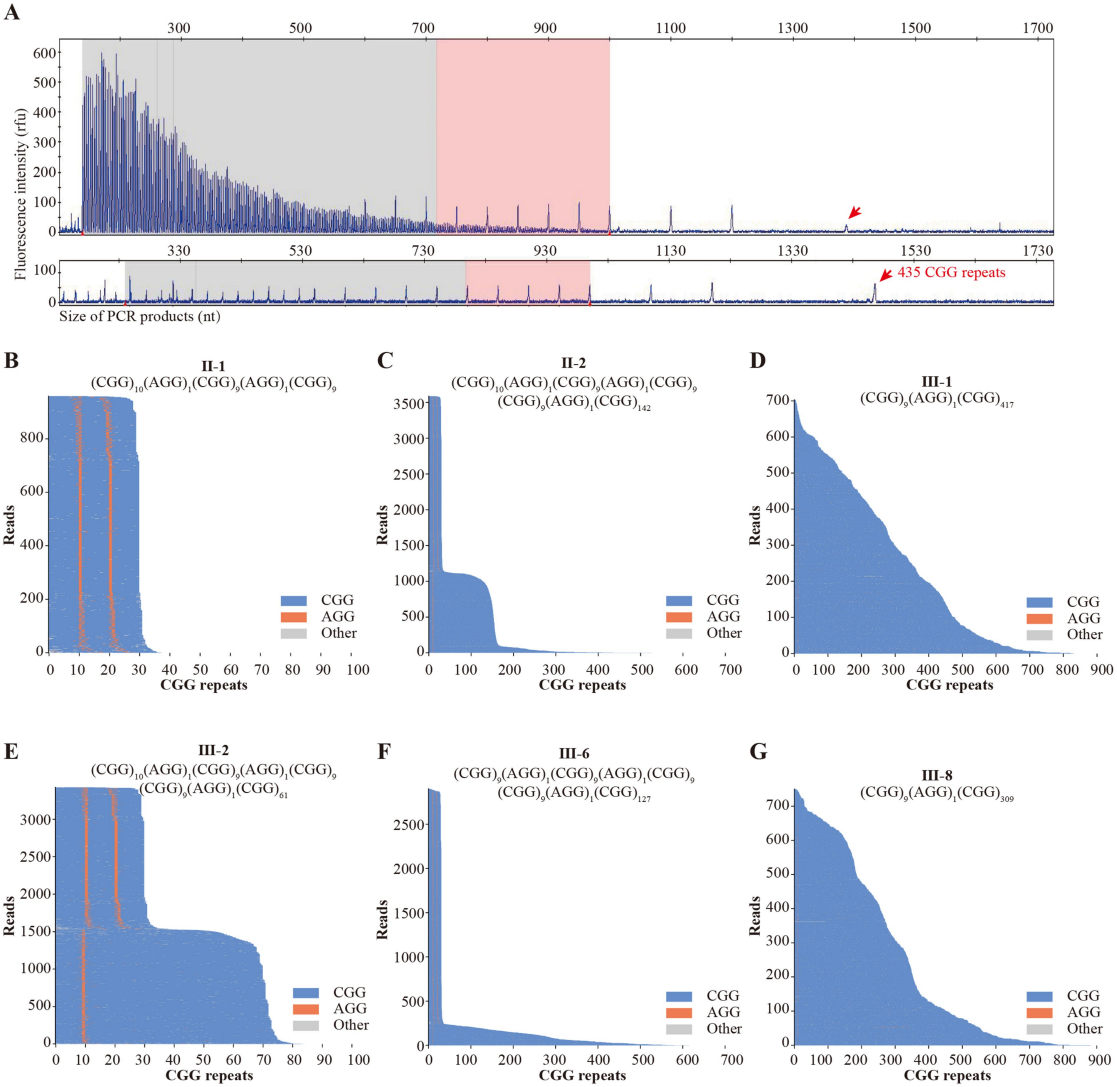
The genetic testing results of this pedigree. **(A)** TP-PCR/CE results of proband III-1. The top result is from repetition primer amplification, while the bottom is from full-length primer amplification. The CAFXS results of II-1 **(B)**, II-2 **(C)**, III-1 **(D)**, III-2 **(E)**, III-6 **(F)**, and III-8 **(G)** were shown in the waterfall figure. TP-PCR/CE: triplet-repeat primed PCR with capillary electrophoresis (CE); CAFXS: comprehensive analysis of fragile X syndrome.

Pedigree analysis was repeated, and combined with negative WES and karyotyping results (with no consanguineous marriage reported), the inheritance pattern did not fit classical autosomal dominant/recessive or X-linked dominant/recessive models. Given that FXS is the most prevalent inherited ID with X-linked semidominant inheritance, TP-PCR/CE was performed on proband III-1’s peripheral blood DNA. Results confirmed proband III-1 as an FXS full-mutation patient (CGG repeats >200; Figure 2A).

### 3.4 FXS is the genetic cause of ID for this family

Counselee III-2 was informed of the test results, along with the advantages and disadvantages of TP-PCR/CE and CAFXS—two current diagnostic methods for FXS. To comprehensively evaluate the risk of FXS in her offspring, counselee III-2 opted for CAFXS. Per this decision, peripheral blood samples from relevant family members were collected for CAFXS, aiming to comprehensively assess the genetic status of both counselee III-2 and this pedigree.

CAFXS testing revealed that the father (II-1) of proband III-1 and counselee III-2 had a normal genotype (Figure 2B). At the same time, their mother (II-2) was a pre-mutation carrier (Figure 2C). Proband III-1 was confirmed as a full-mutation individual (Figure 2D). The counselee III-2 was a pre-mutation carrier (Figure 2E). Additionally, II-4 and II-6 were identified as female pre-mutation carriers (Table 1). Their ID offspring (III-6 and III-8) were identified as full-mutation individuals (Figures 2F,G).

**Table 1:** The CAFXS results of this pedigree.

No.	Gender	CGG repeats	Arrangement of CGG and AGG interruptions
II-1	Male	30	10A9A9^a^
II-2	Female	30/152	10A9A9; 9A142
II-3	Male	30	10A9A9
II-4	Female	29/117	9A9A9; 9A107
II-6	Female	29/137	9A9A9; 9A127
III-1	Male	427	9A417
III-2	Female	30/71	10A9A9; 9A61
III-3	Female	30	10A9A9
III-4	Female	30	10A9A9
III-6	Female	30/283	10A9A9; 9A273
III-8	Male	319	9A309

^a^ 10A9A9 denotes (CGG)10(AGG)1(CGG)9(AGG)1(CGG)9.

Detailed CGG repeat counts and AGG insertion patterns are presented in Table 1. The most common CGG repeat pattern in this pedigree was 10A9A9, abbreviated for (CGG)₁₀(AGG)₁(CGG)₉(AGG)₁(CGG)₉. Dynamic CGG repeat expansions in all full-mutation patients and pre-mutation carriers occurred after the first AGG insertion. Offspring of pre-mutation carriers II-4 and II-6 both developed into increased CGG repeats, progressing to full-mutation. In contrast, pre-mutation carrier II-2 had offspring with variable repeat dynamics: one developed a full-mutation (427 repeats), one had a reduced pre-mutation (71 repeats, III-2), and the other two inherited her normal chromosome X.

### 3.5 Laboratory and imaging findings found no current FXPOI in the counselee

Baseline hormonal profiling of counselee III-2 revealed serum levels of estradiol (20.06 pg./mL), luteinizing hormone (9.03 mIU/mL), progesterone (0.6 ng/mL), and FSH (5.14 mIU/mL). Pelvic ultrasonography showed uterine dimensions of 43 mm × 38 mm × 33 mm, with left ovarian size of 30.8 mm × 19.2 mm and right ovarian size of 35.9 mm × 13.2 mm.

## 4 Discussion

FXS, the leading cause of XLID, arises predominantly from CGG repeat expansions in the 5’UTR of *FMR1* (Monaghan et al., 2013; Spector et al., 2021). Pre-mutation carriers of *FMR1* often exhibit no clinical symptoms during reproductive years, yet carry a risk of transmitting full-mutation to offspring (Monaghan et al., 2013). In the pedigree here, female pre-mutation carriers in the II generation showed no FXS-associated manifestations, yet three full-mutation cases with ID were identified in the III generation. This highlights the critical role of FXS pre-mutation carrier screening in informing reproductive counseling, thereby reducing FXS incidence and enhancing the proportion of healthy offspring. Through comprehensive family history collection and multiple genetic testing, FXS was identified as the genetic etiology of ID in the investigated pedigree. Furthermore, insights into FXS genetic diagnosis and counseling strategies are discussed here.

FXS is the most common inherited cause of ID (Ciaccio et al., 2017). Its phenotypic spectrum is highly complex, comprising not only neurodevelopmental abnormalities underlying ID but also multi-system impairments (Ciaccio et al., 2017). In full-mutation individuals, ID is the typical phenotype, or is accompanied by autism and attention-deficit/hyperactivity disorder (Spector et al., 2021). With advancing age, pre-mutation carriers of both sexes are at risk for FXTAS, while females face additional risk of FXPOI (Hagerman and Hagerman, 2016; Jacquemont et al., 2007; Shelly et al., 2021). The additional X chromosome in females typically results in milder phenotypes of both full-mutation individuals and pre-mutation carriers compared to males (Spector et al., 2021). In our study, full-mutation female III-6 manifested with milder ID compared to males III-1 and III-8, also likely attributed to a shorter CGG repeat length (283 vs. 427 and 319). Notably, an asymptomatic full-mutation female with 280 CGG triplets has been reported (Hung et al., 2019). The high phenotypic heterogeneity of FXS complicates genetic counseling by limiting specific evidence for rapid genetic mapping. Given its relatively high prevalence, preconception and prenatal screening for FXS are crucial for early detection and intervention.

**Figure 3:**
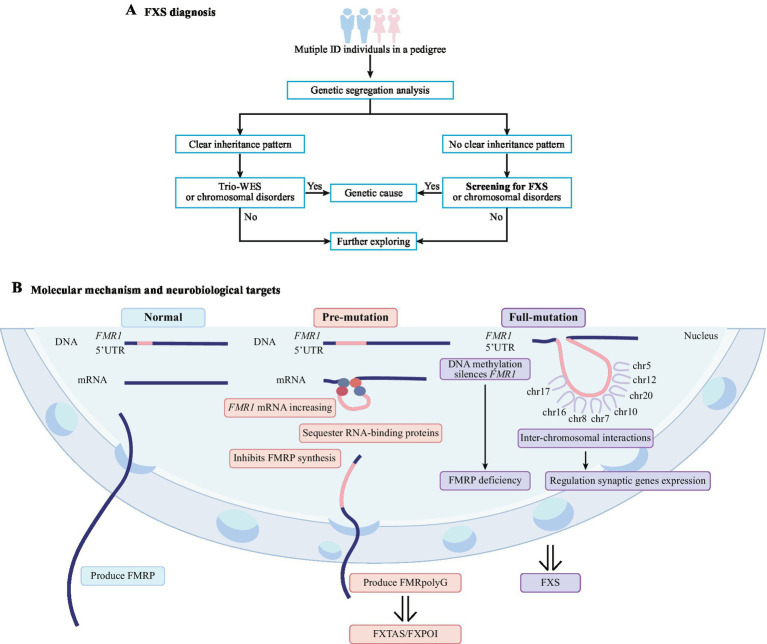
Genetic diagnosis flowchart and molecular mechanisms of fragile X syndrome (FXS). **(A)** Diagnostic process for a pedigree with multiple individuals with intellectual disability (ID) via genetic segregation analysis. **(B)** Pathogenic mechanisms of pre-mutation and full-mutation in the *FMR1* gene. *FMR1*: fragile X messenger ribonucleoprotein 1; FMRP: fragile X messenger ribonucleoprotein; FXTAS: fragile X-associated tremor/ataxia syndrome; FXPOI: fragile X-associated primary ovarian insufficiency.

In this study, the counselee III-2 sought preconception counseling due to her brother (proband III-1) presenting with ID. Initially, only partial pedigree data covering II-1, II-2, III-1, III-2, III-3, and III-4 were provided, with a request for WES. However, WES failed to identify pathogenic variants consistent with the family’s phenotype. Recontacting the counselee for extended pedigree analysis revealed three individuals with ID in the family. Genetic segregation analysis showed that the phenotypic inheritance pattern did not align with classical autosomal or X-linked dominant/recessive models (Figure 3A). Considering FXS is the most common inherited cause of ID and follows an X-linked semidominant inheritance pattern, TP-PCR/CE analysis was performed on proband III-1’s DNA sample, confirming a *FMR1* full-mutation. This case revealed the limitations of WES in FXS diagnosis and emphasizes the indispensable role of comprehensive pedigree data collection in genetic analysis (Figure 3A).

Current FXS diagnostic modalities primarily include Southern blot analysis, TP-PCR/CE, and CAFXS (Spector et al., 2021). Short-read sequencing technologies like WES are inadequate for FXS diagnosis (Spector et al., 2021). The GC-rich CGG repeat tract poses challenges for amplification and accurate mapping, often leading to undetected expansions over 200 repeats, missed mosaicism, and inability to define AGG interruption patterns—key factors in assessing mutation stability (Spector et al., 2021). This explains the failure of WES to identify the genetic etiology in this ID-affected pedigree. The screening value of the above methods was limited by their high costs and prolonged workflows. While TP-PCR/CE and nanopore sequencing shortened testing duration, CAFXS provided the highest diagnostic accuracy, albeit with higher costs and longer turnaround times (Xia et al., 2025). Development of cost-effective, high-accuracy diagnostic methods for FXS could potentially revolutionize current testing paradigms.

To achieve the precise assessment of the *FMR1* gene, CAFXS was employed in this pedigree analysis. CAFXS also confirmed the diagnosis of III-1 as a full-mutation individual. While prior studies indicate a near 100% risk of full-mutation transmission when female pre-mutation carriers have ≥140 CGG repeats (Nolin et al., 2003), counselee III-2 was identified as a pre-mutation carrier with 71 repeats, fewer than her mother II-2 (152 repeats). Despite this intergenerational reduction in CGG repeat length, pre-mutation carriers II-2, II-4, and II-6 each transmitted full-mutation to their offspring. These findings demonstrate that bidirectional CGG repeat dynamics (expansion and reduction) occur during genetic transmission, complicating predictive modeling for offspring risk. Advancing mechanistic insights into FXS could unlock novel strategies to refine transmission risk prediction and reduction.

The primary pathogenesis of FXS arises from dynamic expansions of CGG trinucleotide repeats in the *FMR1* 5’UTR (Hagerman and Hagerman, 2021) (Figure 3B). In full-mutation individuals (CGG repeats ≥200), local DNA methylation silences *FMR1*, driving FMRP deficiency and FXS (Hagerman and Hagerman, 2021; Malachowski et al., 2023) (Figure 3B). The CGG repeat length further influences FXS pathogenesis by modulating inter-chromosomal interactions to regulate long synaptic genes expression (Malachowski et al., 2023) (Figure 3B).

Pre-mutation carriers (55 < CGG repeats <200) exhibit ≥5-fold *FMR1* mRNA elevation, driving degenerative disorders like FXTAS and FXPOI via RNA toxicity or repeat-associated non-AUG (RAN) translation (Hagerman and Hagerman, 2021). Expanded CGG repeats undergo RAN translation to produce FMRpolyG, a uORF that inhibits FMRP synthesis (Sellier et al., 2017) (Figure 3B). FMRpolyG interacts with LAP2b to disrupt nuclear lamina structure, induce neuronal cell death, and form toxic aggregates linked to motor deficits and shortened lifespan in transgenic mice, thereby driving FXTAS pathogenesis (Rodriguez et al., 2020; Sirois et al., 2024).

FMRpolyG also contributes to FXPOI pathogenesis by disrupting granulosa cell mitosis, via reduced mTOR phosphorylation and nuclear architecture perturbation, thereby accelerating antral follicle atresia, diminishing anti-Müllerian hormone (AMH) production, and activating primordial follicles prematurely (Rose and Brown, 2020; Shelly et al., 2021). Furthermore, expanded CGG-repeat RNA forms stable intranuclear foci that sequester RNA-binding proteins (e.g., FUS, PA2G4, TRA2β), disrupting their regulation, and driving granulosa cell death independently of FMRpolyG expression (Rosario et al., 2022) (Figure 3B). FXPOI risk peaks at 59–99 CGG repeats, declining at 100–200 (Pastore et al., 2019). Pre-mutation carriers require higher doses of FSH for ovarian stimulation but exhibit similar ovarian response and pregnancy rates compared to full-mutation individuals (Ranisavljevic et al., 2020). FXPOI patients with *FMR1* pre-mutations exhibit a mean menopause onset 5 years earlier than non-carriers (Shelly et al., 2021). The allelic score integrating CGG length and AGG patterns fails to independently predict amenorrhea age in *FMR1* pre-mutation carriers (Rodrigues et al., 2024). Thus, predictive assessment of FXPOI risk allows for timely recommendations on cryopreserving reproductive materials (e.g., embryos, oocytes, ovarian tissue), providing substantial fertility preservation benefits to female FXS pre-mutation carriers intending to delay pregnancy (La Marca and Mastellari, 2021). However, the unclear mechanism of CGG repeat instability hinders the prediction of FXPOI risk in pre-mutation carriers or full-mutation transmission to offspring. Additionally, limited FXPOI knowledge among women’s healthcare providers may delay *FMR1* testing for POI patients, underscoring the need for enhanced education on FXPOI-FXS associations and integrated genetic counseling to enable timely screening and risk assessment (Singleton et al., 2025). Large-scale cohort studies are therefore critical for establishing clinical patterns and providing evidence for genetic counseling and reproductive guidance.

In this pedigree, both pre-mutation (II-2, II-4, and II-6) and full-mutation (III-6) females achieved successful natural pregnancies. The primary concern of counselee III-2 (a pre-mutation carrier female) centered on the risk of ID in offspring. While higher maternal CGG repeat lengths correlate with increased offspring full-mutation risk, approaching 100% for female pre-mutation carriers with CGG repeats≥140 (Nolin et al., 2003). Even more, maternal AGG interruptions in the *FMR1* gene mitigate the risk of offspring developing FXS (Yrigollen et al., 2012). However, the inherent unpredictability of dynamic CGG repeat expansions is strikingly evident. Maternal carrier II-2 (152 CGG repeats) gave birth to four children: one with full-mutation (III-1, 427 repeats), one with reduced pre-mutation (III-2, 71 repeats), and two with normal genotype (III-3 and III-4), indicating unpredictable expansion dynamics in this pedigree. Notably, III-2’s CGG repeat length (71) decreased from the maternal 152, complicating the risk prediction. The full-mutation individual III-6 (283 repeats) here exhibits mild ID, and prior reports describe asymptomatic females with 280 CGG repeats. These findings highlight the limitations of using repeat length alone to predict full-mutation risk or ID severity in III-2’s offspring. To mitigate full-mutation transmission risk for counselee III-2, available strategies include prenatal diagnosis after natural conception or preimplantation genetic testing for assisted reproductive technologies (Cohen et al., 2021; Hung et al., 2019; Persico et al., 2023; Sonigo et al., 2021).

## 5 Conclusion

FXS underlies ID in this pedigree, highlighting the critical clinical importance of screening ID families with atypical inheritance patterns. Such screening aids in preventing diagnostic oversights related to FXS and decreases the incidence of the syndrome by offering genetic and reproductive counseling to individuals who are pre-mutation carriers.

## Data Availability

The datasets presented in this article are not readily available because of privacy restrictions. Requests to access the datasets should be directed to the corresponding author.
